# Twenty-Four-Hour Heart Rate Is a Trait but Not State Marker for Depression in a Pilot Randomized Controlled Trial With a Single Infusion of Ketamine

**DOI:** 10.3389/fpsyt.2021.696170

**Published:** 2021-07-29

**Authors:** Carmen Schiweck, Erika Lutin, Walter De Raedt, Olivia Cools, Violette Coppens, Manuel Morrens, Chris Van Hoof, Elske Vrieze, Stephan Claes

**Affiliations:** ^1^Department of Neurosciences, Psychiatry Research Group, KU Leuven-University of Leuven, Leuven, Belgium; ^2^Department of Psychiatry, Psychosomatics and Psychotherapy, Goethe University, Frankfurt am Main, Germany; ^3^Electrical Engineering, ESAT-MICAS Department, KU Leuven, Leuven, Belgium; ^4^Imec, Leuven, Belgium; ^5^Collaborative Antwerp Psychiatric Research Institute, University Antwerp, Antwerp, Belgium; ^6^Department of Psychiatry, University Psychiatric Centre Duffel, Duffel, Belgium; ^7^OnePlanet Research Center, Wageningen, Netherlands; ^8^Department of Psychiatry, University Psychiatric Centre KU Leuven, Leuven, Belgium

**Keywords:** heart rate, depression, heart rate variability, circadian rhythm, ketamine, biomarker

## Abstract

**Background:** Abnormalities of heart rate (HR) and its variability are characteristic of major depressive disorder (MDD). However, circadian rhythm is rarely taken into account when statistically exploring state or trait markers for depression.

**Methods:** A 4-day electrocardiogram was recorded for 16 treatment-resistant patients with MDD and 16 age- and sex-matched controls before, and for the patient group only, after a single treatment with the rapid-acting antidepressant ketamine or placebo (clinical trial registration available on https://www.clinicaltrialsregister.eu/ with EUDRACT number 2016-001715-21). Circadian rhythm differences of HR and the root mean square of successive differences (RMSSD) were compared between groups and were explored for classification purposes. Baseline HR/RMSSD were tested as predictors for treatment response, and physiological measures were assessed as state markers.

**Results:** Patients showed higher HR and lower RMSSD alongside marked reductions in HR amplitude and RMSSD variation throughout the day. Excellent classification accuracy was achieved using HR during the night, particularly between 2 and 3 a.m. (90.6%). A positive association between baseline HR and treatment response (*r* = 0.55, *p* = 0.046) pointed toward better treatment outcome in patients with higher HR. Heart rate also decreased significantly following treatment but was not associated with improved mood after a single infusion of ketamine.

**Limitations:** Our study had a limited sample size, and patients were treated with concomitant antidepressant medication.

**Conclusion:** Patients with depression show a markedly reduced amplitude for HR and dysregulated RMSSD fluctuation. Higher HR and lower RMSSD in depression remain intact throughout a 24-h day, with the highest classification accuracy during the night. Baseline HR levels show potential for treatment response prediction but did not show potential as state markers in this study.

**Clinical trial registration:** EUDRACT number 2016-001715-21.

## Introduction

Research over the past 50 years has posited an association between depression and aberrant autonomic nervous system (ANS) activity (1). Significantly increased heart rate (HR) and low levels of heart rate variability (HRV) as surrogate markers for ANS functioning seem to support this position. The HR/HRV changes in individuals with major depressive disorder (MDD) have been described for both short recordings and psychological exposures, demonstrating robustness across several conditions (2). While these changes are usually tested in relatively short time intervals (i.e., minutes), pronounced alterations in patients with MDD have also been described for cardiac circadian rhythm variations. Compared to “normal” circadian variation in the healthy population, early research reports robustly reduced amplitudes of HR/HRV indexes in depression using 24-h recordings (3–7). It is noteworthy that altered amplitude of circadian rhythm is not only limited to cardiac parameters but has also been observed for other measures such as plasma cortisol, norepinephrine, or body temperature (8), emphasizing that these alterations do not merely reflect altered activity patterns but are a wide reaching and consistent feature of MDD.

More recently, the application of cardiac parameters as potential, non-invasive state markers for depression has been discussed. The relevance for such markers would be highly relevant for early warning mobile applications, but current real-life studies for such applications are still sparse. Among the first to experimentally demonstrate the potential of cardiac parameters as state markers, Bylsma et al. (9) revealed normalization of cardiovascular parameters in remitted patients toward controls and compared to currently depressed patients in a cross-sectional stress paradigm (9). However, treatment paradigms investigating HRV indexes as potential state markers have shown mixed results: While some found normalization toward controls after antidepressant treatment for at least one HRV index (10–13), others suggest HR/HRV is better used as a trait rather than state marker for depression (3, 14–16). Nonetheless, usefulness as a predictive marker for treatment response prediction was achieved in some of these reports. Notably, the study paradigms used in the research described above focused on short-term recordings during specific times of day or do not mention a time of recording. Yet, in light of the established circadian rhythm differences in MDD, circadian rhythm variations may yield more insight and potentially more precise results as biomarkers. Indeed, Saad et al. (17) have shown that night recordings in particular might be useful markers for depression (17). Recordings during sleep may constitute a more robust biomarker, given the absence of conscious neural modulation and external, potentially stressful stimuli or movement that could obscure HR measurements. Given the new technical advances, long-term, real-life monitoring with unobtrusive hardware may therefore be a less-biased measure to assess biomarkers for depression. Real-life home monitoring makes it possible to correct movement artifacts and use circadian rhythm variations as selective markers rather than as cofounding element encountered in depression.

Regular antidepressant treatments can take weeks to months before achieving the desired effect (18). Side effects of treatment include, among others, weight gain or loss (19), nausea, and altered activity/energy levels—all of which may have an impact on HR/HRV indexes. Treatment with the recent, rapid-acting antidepressant ketamine yields a unique paradigm for testing whether changes in mood are associated with changes in HR or HRV, without significant changes of other covariates: Within a short period of time (usually 24–48 h), drastic improvements in mood can be seen in a majority of patients with depression (20) with relapse occurring within 7 days after administration of ketamine (21). If HR and HRV indexes can be directly correlated to changes in mood, treatment with ketamine may reveal such associations.

The aim of this study is three-fold: First, we aim to confirm circadian rhythm abnormalities in a well-defined sample of patients with treatment-resistant depression (hereafter referred to as “patients”) and matched controls using wearable technology, with potential for clinically applicable remote monitoring. Second, we aim to examine the potential of HR and root mean square of successive difference (RMSSD), with particular focus on the night period, as a biomarker to correctly categorize patients and controls. Third, we aim to assess HR and RMSSD before and after antidepressant treatment with a single dose of ketamine or placebo to assess its applicability for treatment response prediction and as a state marker of depression. We hypothesize a different circadian rhythm between depressed and control groups, as evidenced by a lower amplitude, and hypothesize that real-life psychophysiology monitoring can be used to accurately distinguish patients with MDD from controls. Lastly, we hypothesize that cardiovascular parameters predict treatment response to ketamine and improve with mood.

## Methods

This study was approved by the Ethics Committee Research UZ/KU Leuven, and written informed consent was obtained from all participants prior to any study-related procedure.

### Participants

For this report, 32 participants were available. Psychophysiological data was available for 16 adult patients satisfying diagnostic criteria for MDD, without psychotic features [DSM-IV 296.22, 296.23, 296.32, or 296.33, *n* = 13, or bipolar disorder (BPD) type I or II in a depressed episode without recent (hypo) manic episodes: DSM-IV 296.89] as per the Diagnostic and Statistical Manual of Mental Disorders–Fourth Edition Text Revised (DSM-IV-TR; *n* = 3), and 16 sex-matched controls with comparable age was available from the FEEDBACK Trial (ethical registration number S59102, EU Clinical Trials registration number 2016-001715-21). The trial was stopped after the foreseen recruitment period (see CONSORT flow diagram in Supplementary Figure 1). Depression diagnosis was confirmed by correspondence with treating physicians and/or psychiatrists and independent validation using the Dutch Version of the Mini International Neuropsychiatric Interview 5.0 (MINI) (22) as diagnostic interview by a trained psychologist or psychiatrist. To be included, patients (with MDD and BPD) needed to have at least moderate depressive symptoms as determined by a depression score of 17 or above on the Hamilton Rating Scale for Depression (HRSD) (23), must have had at least two courses of different antidepressant medication, or for BPD 2 courses of antidepressant/lithium, and were currently not taking anti-inflammatory medication (e.g., no systematic use of corticosteroids, antipyretics, antibiotics, or non-steroidal anti-inflammatory drugs on a regular basis; other drugs were evaluated on a case-to-case basis). Patients with BPD needed to be taking mood-stabilizing medication 4 weeks prior to and throughout the study. Patients and controls had to be free of cardiovascular disease (additionally confirmed with an ECG), show no signs of hypertension (diastolic blood pressure <90 mmHg), and were free from a history of drug abuse. Concomitant antidepressant medication was allowed but limited to four concurrent psychotropic medications and had to be kept on a stable dose for at least 30 days before the start of the study. Patients had to be naïve to ketamine treatment in order to participate. Demographic data and comparisons can be found in Table 1 and Supplementary Table 1.

**Table 1:** Demographic data.

	**Controls**, ***n*****=16**		**Patients**, ***n*****=16**		***t*-Value/*X*^2^**	***p*-Value**
	**Mean**	***SD***	**Mean**	***SD***		
Age	43.62	12.57	46	12.29	−0.54	0.593 ns
BMI	24.61	4.24	30.48	5.24	−3.48	0.002**
HRSD	2.12	2.0	21.88	4.26	−16.81	<0.001***
QIDS	2	2.39	18,50	3.08	−16.93	<0.001***
Smoking	12%		19%	–	0.237	0.626 ns
Sex female	69%	–	69%	–	0	1 ns
HR (bpm)	62.44	5.33	77.79	9.53	−5.62	<0.001***
RMSSD (ms)	69.64	20.5	38.88	14.34	4.96	<0.001***
Std. ACC	0.05	0.01	0.03	0.01	6.39	<0.001***
**Antidepressant medication**			***n***	**%**		
SSRI/SNRI/SARI	–	–	11	69%	–	–
TCA/TECA	–	–	5	31%	–	–
Benzodiazepines/Atypical/Other	–	–	14	88%	–	–
Unmedicated			0	0%		

Differences are indicated for healthy controls and patients, matched for sex and of similar age. For wearable data of patients, pre- and post-treatment phases are taken into account. BMI, body mass index; HRSD, Hamilton Ratings Scale for Depression; QIDS-SR, Quick Inventory of Depressive Symptomatology Self-Rated version; HR, heart rate at baseline; RMSSD, root mean square of successive differences at baseline; Std ACC, unfiltered standard deviation of the activity index. * P < 0.05; ** P < 0.01; *** P < 0.001; ns, not significant. Bold values indicate significant results.

### Questionnaires

Depressive symptom severity was determined using the HRSD 17-item version, and participants completed the Quick Inventory of Depressive Symptomatology (self-rated version). In addition, the Depression Anxiety and Stress Scale (DASS) was administered. During the psychophysiological data collection phase, participants were also invited to indicate whether they had subjectively slept well (yes/no) every morning before 10 a.m., using a questionnaire on a smartphone.

### Study Design and Ketamine Treatment

**Figure 1:**
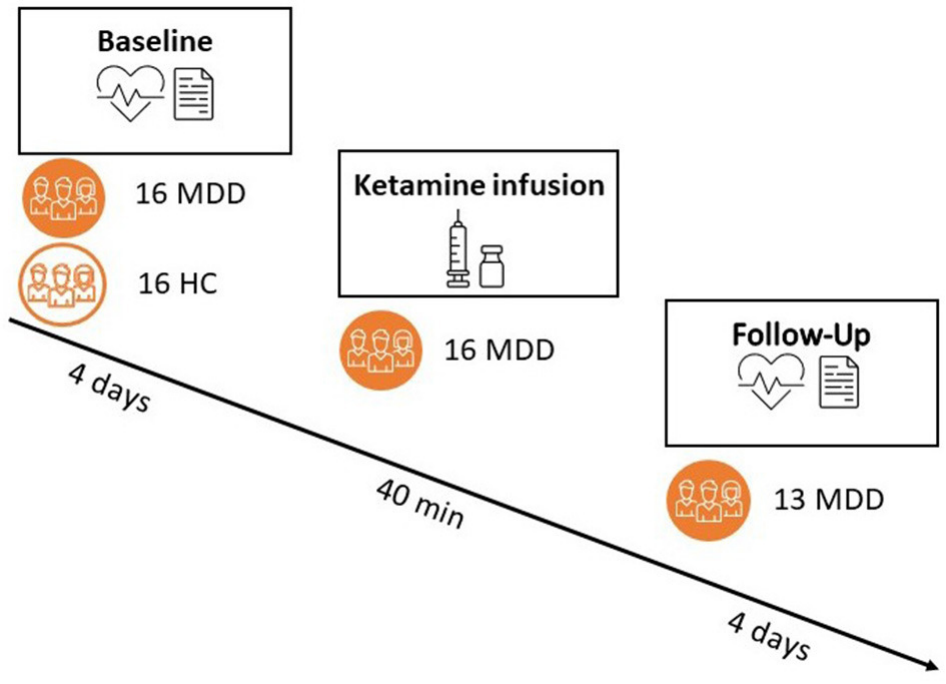
Overview of the study design. Both patients and controls completed a pre-phase (“baseline”) of 6 days, with a portable ECG recording during the first 4 days. Only patients proceeded to a treatment with either ketamine (66%) or placebo (33%) and a subsequent follow-up phase of 6 days (out of which 4 days with psychophysiological recording). Responder status was assessed 24 h post-ketamine administration.

The current study was a two-center, multiple-phase, randomized controlled trial with parallel arms of ketamine/placebo treatment in diagnosed patients with MDD and incomplete response to antidepressant therapy. The study consisted of a baseline phase including collection of questionnaires, blood samples (not reported), 4 days of psychophysiological (ECG) at home, and 6 days of ecological momentary assessment sampling (not reported). Second, there was a treatment intervention that consisted of one placebo-controlled administration of intravenous (R,S) ketamine in a 2:1 ratio of ketamine/placebo. The study medication/placebo was prepared per protocol by the designated pharmacies. Subjects with MDD were randomized to one single administration of intravenous ketamine (0.5 mg/kg, 40 min infusion) or an equal amount/duration of saline solution. The infusion was identical for both products, performed with an automatic infusion pump under supervision of an anesthesiologist. The treatment phase was followed by a follow-up phase identical to the baseline measurements (4 days of psychophysiological recording). We allowed for a 4-h recovery period after ketamine administration before starting the recording in order for the acute effects of ketamine to dissipate. Healthy controls only completed the baseline assessment. An overview of the study design can be found in Figure 1.

### Randomization Procedure

Upon entry into the study (screening), patients were attributed the next number in the list of available study participant numbers, which was a three-number code generated at random. This number was matched to a randomization code for ketamine/placebo that had been created and matched by a third party (Johnson and Johnson pharmaceuticals) with a 1:2 allocation ratio (placebo/ketamine) per block of 20 individuals. The codes were directly transmitted to the pharmacy and were stored in a folder inaccessible to any person other than the designated study pharmacist. All study employees directly or indirectly involved with the patient (except for the pharmacy) had no access to randomization codes at any time, until sealing of the electronic case reporting form at the end of the complete study.

### Treatment Response to Ketamine/Placebo

For the purpose of this study, we distinguished two groups: non-responders, defined as <30% reduction of HRSD (non-responders) between pre-assessment and 24 h post-administration and a second group of “responders,” consisting of partial responders and full responders [change score of at least 30% as proposed by Irwin et al. (24) and 50%; e.g., (25), respectively]. Additionally, to achieve more power, we used change scores on the HRSD from baseline to 24 h post-ketamine as secondary outcome point.

### Devices

The participants were provided with a wireless chest patch “BioTel Research ePatch” (Biotelemetry, Horsholm, Denmark). The chest patch measures a 2-lead ECG at 256 Hz and acceleration (ACC) at 32 Hz. The participants wore the patch both day and night. The participants were asked to wear the sensor for the entire recording period. Exceptions were permitted if sensors were experienced as disagreeable during the night (two occurrences, one patient and one control during one night).

### Feature Calculation

Two features were calculated from the ECG signal: the mean HR and the root mean square of the successive differences (RMSSD) in RR peaks. We selected RMSSD as it has been shown to be an indicator for cardiac vagal modulation and thus may provide information regarding differences in parasympathetic ANS activation. Choosing high-frequency heart rate variability (HF-HRV) as an indicator for cardiac vagal activation is less suitable for our data since we were unable to accurately address respiratory activation. Both features were calculated in a window of 5 min (overlap of 4 min), which is the minimum window required to calculate HRV features (26). The activity level of the participants was calculated as the standard deviation of the magnitude of acceleration as derived from the accelerometer of the ECG patch. In accordance with the ECG features, activity levels were calculated in windows of 5 min with 4-min overlap.

### Quality and Activity Filtering

The quality of ambulant physiological recordings may be affected by artifacts from motion and poor sensor attachment. Therefore, a quality indicator was assigned to every 10-s segment of the signal. The quality indicator is based on research published by Orphanidou et al. (27). In order to qualify as “high quality,” two steps are passed: In the first step, the algorithm performs a beat detection and assesses the derived output for the satisfaction of three criteria: (a) a physiologically plausible HR (e.g., between 40 and 180 bpm), (b) a criterion establishing a maximum distance between adjacent RR peaks (3 s), and (c) a cut-off value for the ratio of minimum and maximum beat-to-beat interval length for each identified segment (>2.2). These criteria have shown to be excellent indicators for detection of artifacts (27, 28). If all three rules are satisfied, the algorithm proceeds to the second step, consisting of adaptive QRS template matching. If the segment is irregular in morphology, it is assumed to be affected by an artifact, resulting in a bad quality label (0). In case of a high level of regularity, the segment is reported as good quality (1). In addition to the algorithm, random checks of 10 segments (5 high- and 5 low-quality segments) were verified manually using KUBIOS software 2.0, which agreed with the algorithm. The resulting data was filtered using the QI. All 5-min windows with an average QI below 0.8 were excluded. In addition, following Smets et al. (28), all segments of high activity (std ACC > 0.04) were excluded as well in order to obtain a clear result in absence of the confounding effect of physical activity. Lastly, all retained windows were averaged into either hourly measurements or blocks of 6 h depending on the analysis.

### General Activity Index

To assess general activity levels of participants, we used the standard deviation of the overall magnitude of the activity during the entire assessment period, unfiltered for high activity, as an indicator for overall activity in patients and controls.

### Statistical Analysis

All statistical analysis was performed in R version 3.6.1. Graphical representations were performed with GraphPad Prism version 8.1.1 and Python version 2.7. Data analysis was performed on all available data with sufficient quality and filtered for high activity levels as described in the *Feature calculation* section. Due to slight deviations for model assumptions, data was log transformed.

In a linear mixed model with random intercept per subject, we predicted ECG outcome variable (HR/HR variability indexes) with the independent variables group, age, sex, body mass index (BMI), and overall activity index. As there are well-known circadian effects of HR and HR variability, we also introduced the first (e.g., per 24 h) and second (e.g., per 12 h) harmonic regression coefficient for time as described in the literature (29). The final model was selected using backwards stepwise regression (model selection based on *p*-value). Secondly, because we were interested in distinction capacity at night, we tested the average data of day (12:00–18:00 h) and night (00:00–06:00 h) periods for classification performance using a binary logistic regression with leave-one-out cross-validation [caret package in R (30)]. Additionally, we repeated the analysis for the hour block with identical activity patterns in both groups.

To determine the effects of treatment on the link between cardiovascular parameters and mood, we conducted two analyses within the MDD patients. First, we assessed whether baseline HR/HRV was different between responders and non-responders for the entire phase. Associations were tested with Spearman's rank correlation between baseline HR/RMSSD and change scores on the HRSD (i.e., the change of depressive symptomatology between baseline and day 9). Second, we ran a linear mixed model including treatment phase (pre- or post-treatment) into the model.

Lastly, since we were not aware of any study design that could be used to estimate effect sizes, we also set out to explore the effect size for this kind of analysis and thus estimate the number of participants needed to reach sufficient power. For this purpose, we simulated a dataset based on expected estimates for 60 participants using the package SimR (31). Given the difficulty of recruiting patients for the trial and loss of data due to insufficient quality of the recording, we only achieved to recruit 32 people with valid ECG information (see CONSORT Flow diagram in Supplementary Figure 1 for an overview). We therefore also added *post-hoc* power calculation for 32 people, on simulated data (see Supplementary Information 1 for power calculations).

## Results

### Demographics and Physiological Data Description

Sixteen patients were matched with 16 controls of similar age and the same sex. Demographic data can be found in Table 1. No significant differences were present for age and sex, and BMI was higher in the depressed population, as expected. Patients had significantly higher depression scores, higher HR, and lower RMSSD (both *p* < 0.001, see Table 1). Measures of clinician-rated and self-rated depression both indicated a moderately to severely depressed group (23). After filtering for quality and activity as described in the *Methods* section, we retained an average of 37.53 (*SD* 10.39) full hours of physiological recording for controls and 48.43 h (*SD* 16.91) for patients, spread over the entire period of approximately 84 h. For the post-ketamine period, 13 participants had data with sufficient quality to use (2 placebo and 11 ketamine). After filtering for quality and activity as described in the *Methods* section, we retained an average of 54.03 h (*SD* 12.96) in the post-treatment phase.

### Psychophysiological Group Differences in Depression

**Figure 2:**
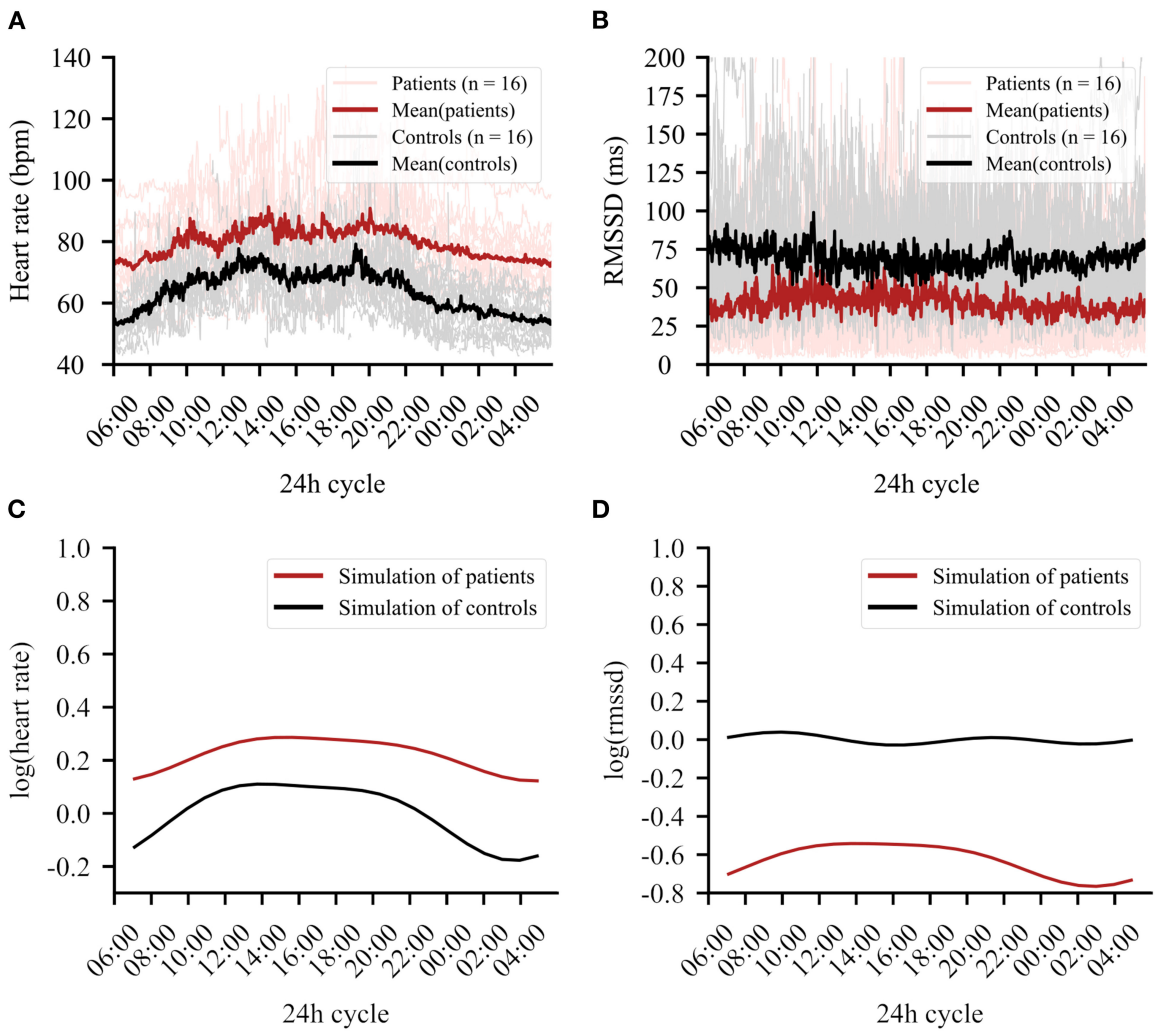
The 24-h cycle averaged over the entire recording period of 4 days. **(A)** Mean heart rate data in beats per minute (bpm) of patients (red) and controls (black). Clear group differences can be observed. **(B)** Mean RMSSD in milliseconds (ms) for patients (red) and controls (black). **(C)** Simulation of the regression line fitted to the data for heart rate (HR). **(D)** Simulation of the regression line fitted to the data for root mean square of successive differences (RMSSD).

A good model fit was achieved for the final model predicting circadian variations in HR (conditional *R* squared: 0.75) and RMSSD (conditional *R* squared: 0.56). The significant main effects for HR included group, age, and circadian rhythm. Subjective sleep quality did not influence HR levels neither during night (*b* = 0.02, *t* = 0.68, *p* = 0.503) nor during day (*b* = −0.01, *t* = −0.33, *p* = 0.744). Significant effects for RMSSD emerged for group, sex, and circadian rhythm. Again, sleep quality did not influence RMSSD levels during the night (*b* = −0.08, *t* = −1.00, *p* = 0.323) and day (*b* = −0.06, *t* = −0.92, *p* = 0.360). A detailed overview of model statistics can be found in Table 2. Figures 2A,B display group differences per time point for HR and RMSSD, respectively. BMI and overall standard activity did not contribute to either model and were therefore not selected in the final models.

**Table 2:** Results of linear mixed models.

	**Estimate**	***t*-Value**	***p*-Value**
**A**			
Group	0.221	6.477	<0.001***
Age	−0.176	−2.908	0.007**
sin[2 × pi/24 × (*t*)]	−0.108	−24.935	<0.001***
cos[2 × pi/24 × (*t*)]	−0.087	−19.835	<0.001***
sin[2 × pi/12 × (*t*)]	−0.033	−7.638	<0.001***
cos[2 × pi/12 × (*t*)]	0.017	5.740	<0.001***
Group × sin[2 × pi/24 × (*t*)]	0.037	6.191	<0.001***
Group × cos[2 × pi/24 × (*t*)]	0.055	9.151	<0.001***
Group × sin[2 × pi/12 × (*t*)]	0.026	4.320	<0.001***
**B**			
Group	−0.632	−5,981	<0.001***
Sex2	0.320	2,806	0.009**
sin[2 × pi/24 × (*t*)]	0.012	0.708	0.479
cos[2 × pi/24 × (*t*)]	−0.008	−0.464	0.642
sin[2 × pi/12 × (*t*)]	−0.025	−2,001	0.046*
Group × sin[2 × pi/24 × (*t*)]	−0.083	−3,403	<0.001***
Group × cos[2 × pi/24 × (*t*)]	−0.077	−3,125	0.002**

(A) Heart rate. Results are presented for significant terms of the linear mixed model predicting heart rate after log transformation, with random intercept. Sine and cosine terms are indicated for 24-h cycles and for 12-h cycles, respectively. Estimates for group and group × harmonic regression terms interactions are indicated for the depression group. (B) RMSSD. Results are presented for significant terms of the linear mixed model predicting RMSSD after log transformation, with random intercept. RMSSD, root mean square of successive differences. *P < 0.05; **P < 0.01; ***P < 0.001; ns, not significant. Bold values indicate significant results.

### Circadian Rhythm Variation Differs per Group

Importantly, a group × circadian rhythm interaction emerged for HR [Table 2(A)] and RMSSD [Table 2(B)], showing altered circadian rhythm variation per group. Figure 2C shows the HR model simulation of circadian rhythms. Figure 2D shows the simulated model for RMSSD. Healthy controls showed higher amplitude in their overall HR circadian rhythm with a prominent drop during night (10 p.m.−6 a.m.) and a steep rise in the morning (6 a.m.−3 p.m.). Circadian variations of healthy controls also showed a 12-h variation, as reflected by an intermediate decrease of HR in the afternoon (3 p.m.). While patients exhibited similar HR circadian rhythm variation, the overall variation was less pronounced, as evidenced by the three significant group × harmonic terms interactions that reflect a reduction of amplitude and variation of circadian rhythm.

For RMSSD, healthy controls showed several smaller fluctuations throughout the day, while the patient group showed a single, slow increase of relatively larger magnitude, reaching a plateau in the early to late afternoon (11 a.m.−6 p.m.). While the increase was relatively larger in the patient group, the general group difference showing lower RMSSD in patients remained intact when testing the two most similar scores between groups (hour block 11–12 smallest difference, *t* = −2.37, *df* = 28.65, *p*-value = 0.025). For a summary of the functions for harmonic regression terms, see Supplementary Figure 2 and Supplementary Table 2.

### Classification of Groups

Since HR and HRV levels are known to be significantly different between patients and controls (32), as a next step, we tested whether we could determine group membership, with particular focus on the pronounced differences in the “night” compared to the “day” period. Although we filtered data on activity such that only low activity data were included, an impact of activity cannot be excluded. We therefore repeated analysis for the hour block showing the lowest activity patterns in both control and depressed groups (hour block 2, see Supplementary Figure 3).

Classification results showed excellent accuracy for the log of HR (day accuracy: 81.25%, kappa: 62.5%; night accuracy: 90.63%, kappa: 81.25%) and RMSSD (day accuracy: 71.88, kappa: 43.75%; night accuracy: 81.25%, kappa: 62.5%). The combination of HR and RMSSD did not outperform HR as predictor alone (day accuracy: 78.13, kappa: 56.25; night accuracy: 87.5%, kappa: 75.0%). During night, 14 controls and 15 patients were classified correctly based on HR. Results were essentially equal for hour block 2 (2–3 a.m.).

### Response Prediction

**Figure 3:**
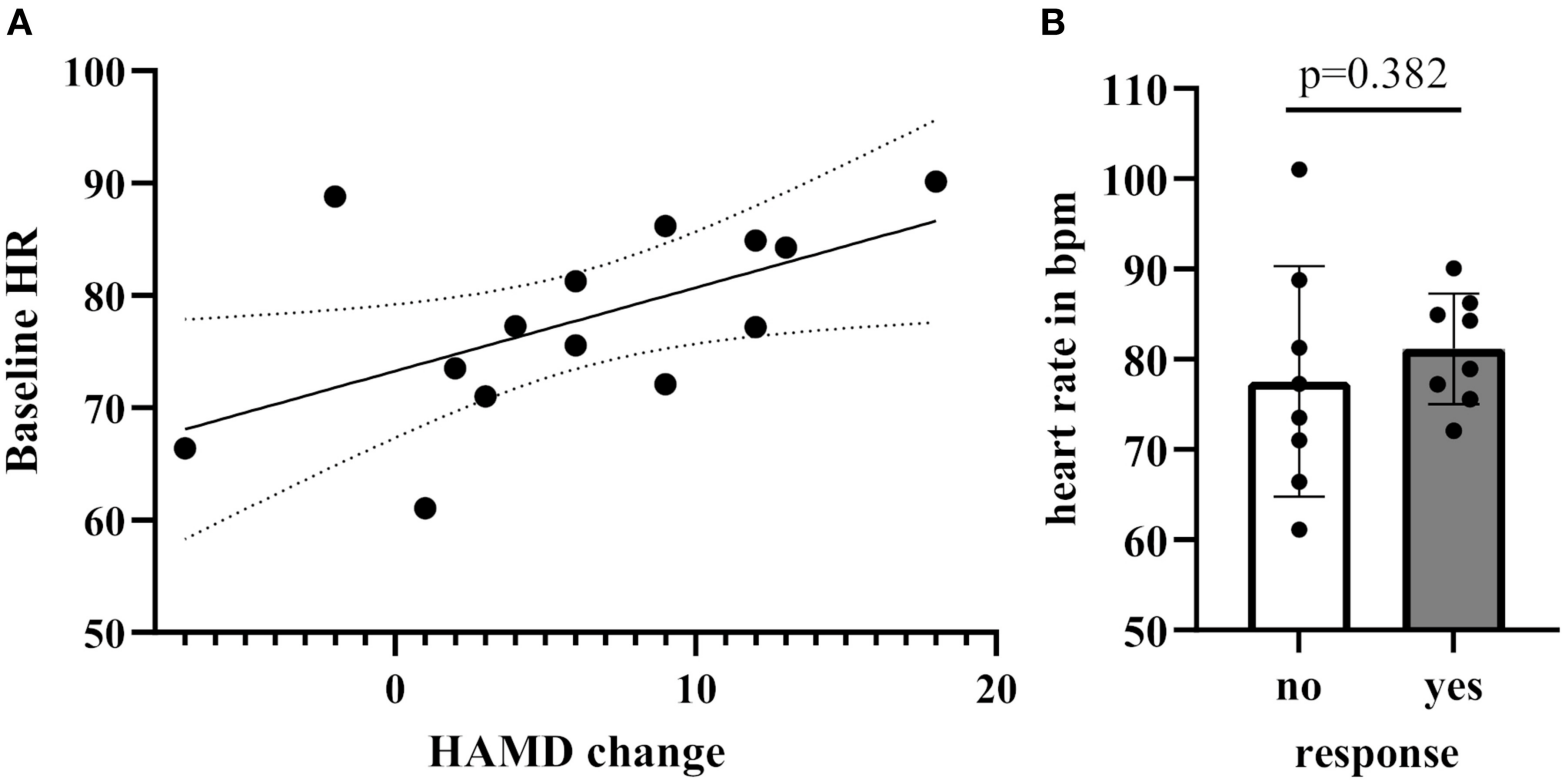
**(A)** Correlation of Hamilton Rating scale for depression score between baseline and 24 h post-treatment with baseline HR levels **(B)** Baseline HR levels grouped by response [no response: <30% reduction of Hamilton Rating Scale for Depression (HRSD), yes response ≥30% reduction on the HRSD].

In order to determine whether treatment response was associated with baseline HR/RMSSD levels, we assessed the difference of HR/RMSSD by responder status. There were no significant differences between responders (*n* = 6) and non-responders (*n* = 7), neither for HR nor RMSSD. A positive correlation between baseline levels of HR and treatment change scores on the HDRS (pre-post scores) emerged after excluding an outlier based on an unlikely value of more than the mean HR plus two times standard deviation (e.g., HR > 99.96) (Spearman's *r* = 0.55, 95% CI: 0.01–0.84, *p* = 0.046; including the outlier Spearman's *r* = 0.31, 95% CI: −0.22–0.70, *p* = 0.24). Analysis by groups (responders/non-responders) revealed no significant differences (difference: 8.1, 95% CI: −2.60–16.60, *p* = 0.152) (Figures 3A,B).

### Treatment Effects

**Figure 4:**
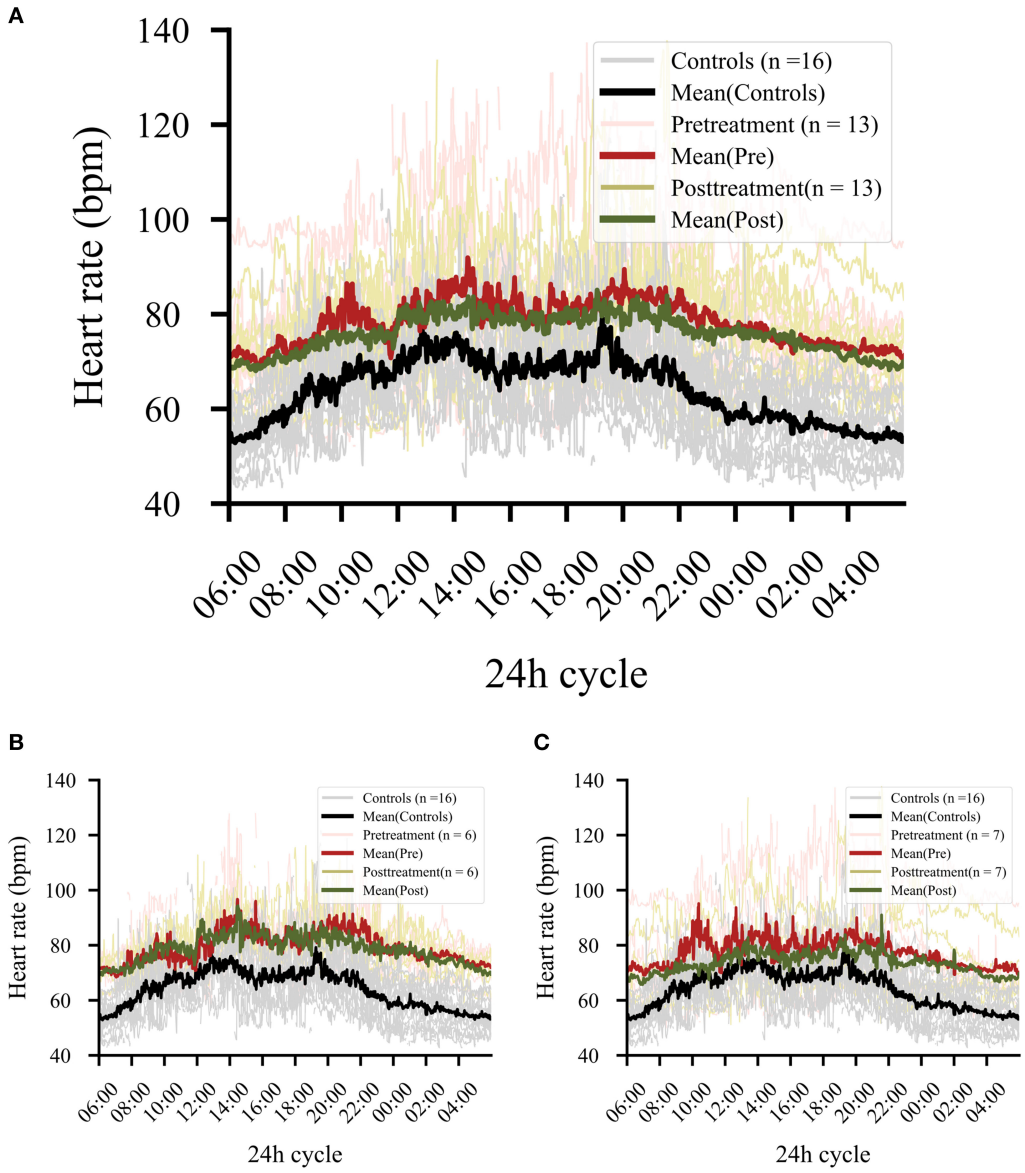
**(A)** Comparison of pre- and post-treatment phase regardless of treatment. Patients (*n* = 13) showed significant drops in heart rate levels in the post-treatment phase (*p* < 0.001). The black line shows the control data collected at baseline, in order to provide a reference for the effect magnitude. **(B)** Effect in all responders independent from treatment (ketamine or placebo). **(C)** Effect in all non-responders independent from treatment (ketamine or placebo).

Thirteen of the 16 patients had data for the post-treatment period. A significant effect emerged comparing pre- and post-phases, showing lower levels of HR in the post-phase (*b* = 0.03, *t* = 5.77, *p* < 0.001) but no significant change of levels occurred for RMSSD (*b* = 0.02, *t* = 1.05, *p* = 0.295). However, a comparison of HR levels in the post-phase with healthy control levels (pre-phase) still showed significant group effects (overall: *b* = 0.16, *t* = 4.41, *p* < 0.001) (Figure 4). A single infusion of ketamine did show a reductive effect of HR in the post-phase (ketamine: *b* = 0.01, *t* = 3.10, *p* = 0.002). Since only two patients from the placebo group had pre- and post-data available, the effect for the placebo group was not explored any further.

## Discussion

In this small dataset of treatment-resistant patients with depression, we show that the well-established association of higher HR and lower RMSSD levels with depression remains intact at all times of a 24-h day, with the strongest differences during the night. The period between 2 and 3 a.m. in particular may be pertinent to assess HR as a biomarker for depression. Patients with depression show a markedly reduced amplitude for HR and less daily fluctuations for RMSSD. However, while baseline HR levels may show potential for treatment response prediction, in these preliminary results, we could not confirm previous studies showing potential for physiological measures as state markers of depression.

### Baseline and Circadian Rhythm Differences in Depression

While short-term recordings have established baseline differences including high HR and low RMSSD in depression (32, 33), only little recent research has focused on cardiac circadian rhythm abnormalities in depression. Early studies have shown that a diagnosis of depression, possibly combined with anxiety, is associated with higher HR and lower HRV in overall 24-h recordings and that HR remains elevated in depressed patients during sleep (6, 34, 35). Taillard et al. (6) showed that the abnormalities in depression particularly involve the HR amplitude indicated by a flattened rise of HR during the day and reduced decrease during the night using the simple cosinor method. Here, we replicate this finding using a customized approach with four different harmonic regression terms and activity-filtered, continuous data. Our results suggest that patients have a markedly reduced amplitude compared to controls and show reduced fluctuation in the 12-h period. Albeit these observations were made in a small group of patients, this could point toward a more rigid response system and/or an exhausted responsiveness to stress in depression.

### Classification of Patients With Depression

We achieved very accurate classification of patients and controls using HR and significant, but slightly diminished, accuracy for RMSSD. This confirmed these indexes as accurate trait markers for depression and identified a particular time window that can be used. Attempts of classification based on short-term physiological recordings have been made before (2, 36), achieving a moderate-to-good accuracy, but classifications based on long-term or night data are limited. Few studies attempted to classify patients based on longitudinal physiological data. Gaetz et al. (34) found a rather poor classification accuracy when using the diurnal physiological measures, but much improved accuracy (70.2%) when using nighttime data. Saad et al. (17) were able to classify depressed patients with sleep abnormalities. The authors showed accurate distinction between patients and controls based on HR and EEG parameters (79.9%) (17). While highly informative, this approach precludes application in a home setting due to recording of an EEG. Here, we establish a very high accuracy by ECG recording filtered for activity; albeit in a small dataset, our classification accuracy suggests great clinical applicability, given the relative ease of application for the procedure.

Classification at night is of particular interest, given that the nighttime is a “resetting point” for the ANS. Contrarily to the day trend for an increase in HR and decrease of HRV, the night phase is usually characterized by a decrease of sympathetic nervous system activity and an increase in parasympathetic activity (37). The higher HR levels and lower RMSSD could indeed point toward a dysfunctional recovery at night. Our results suggest that ECG monitoring, particularly at night, might be a very promising and easily applicable non-invasive biomarker.

### Treatment Response Prediction Based on HR

A biomarker for depression naturally achieves great applicability if it can predict (early) treatment response or can identify depressive states rather than being stable over time. Here, we showed a small positive association of baseline HR with improvement of depressive symptoms after a single ketamine administration, if an outlier was excluded. This may point to a possible effect for HR as a response predictor, but will need to be confirmed, given our low sample size and the high variability of this effect. It should be noted that ketamine itself has effects on cardiovascular output: several studies have reported increased HR during and immediately after ketamine infusion (38, 39). A recent study in healthy volunteers has shown that ketamine can increase HR significantly during a 120-min recording period (39) and generally reduces HF-HRV (40).

Indeed, next to antagonizing the N-methyl-D-aspartic acid receptor, ketamine also affects several other receptors, among which are muscarinic receptors (41), thereby reducing parasympathetic activity. This can result in higher HR and lower levels of measures for parasympathetic activity (such as RMSSD), which has indeed been observed before (40, 42). However, nearly no studies have investigated the cardiovascular effects of ketamine when given in doses relevant for depression treatment, since most studies investigated ketamine as an anesthetic agent. In addition, here we omitted the 4 h following ketamine infusion to allow for the immediate effects to dissipate. Interestingly, a recent study also investigated the use of HR for response prediction to ketamine for depression: Similar to the here-reported results, Meyer et al. (43) have found HR to be significantly higher in responders, supporting a possible predictive value of HR for (ketamine) treatment outcome and warranting further research into the relationship between low-dose ketamine and HR.

The relationship between antidepressant responses and conventional medication may also contribute to the understanding of the association between cardiovascular parameters and mood: Hage et al. (16) showed higher low-frequency (LF) HRV and respiratory sinus arrhythmia in responders, and Glassman et al. (3) showed an association of LF-HRV with improvements in mood. Yet, opposed findings have also been reported: Hartmann et al. (11) found no association of baseline HRV, and Choi et al. (44) reported an association of lower baseline HR with treatment response. A recent large-scale study by Kircanski et al. (45) may shed light on these apparent incongruences: In their 8-week treatment paradigm with venlafaxine, sertraline, or escitalopram, anxious symptomatology in depression played a defining role. While a positive correlation of HR and treatment response emerged for depressed patients with anxious symptoms, this was not true for those without anxious symptoms (45). A similar association emerged for RMSSD, where higher levels of RMSSD in the anxious population were positively associated with response rates. This large-scale study emphasizes the need for a personalized medicine approach, including the individual symptomatology of MDD patients for the selection of functional treatment response predictors.

### Modulation of ANS With Successful Treatment?

Of particular interest is the question if HR and HRV indexes normalize toward healthy controls (i.e., “improve”) in congruence with amelioration of mood and have value as a state marker of depression. Here, we indeed observed that HR levels dropped significantly after treatment. However, this effect could not be associated with treatment response, and no effect emerged for RMSSD. The interaction effect showed that while HR and depression severity were associated before treatment, this was not the case after treatment. It is conceivable that a factor linked to ketamine treatment *per se* or an external factor linked to treatment (e.g., enhanced social interaction and activity) may contribute to a drop in HR levels. Evidently, these conclusions should be treated with caution, given the low sample size, and future studies need to compare the effect between treatment and responder groups.

While we now know that long-term consequences of antidepressant treatment with tricyclic, serotonergic, or noradrenergic agents include an increase of HR and reduction of HRV, as was elegantly and extensively demonstrated by Licht et al. (46) and in a meta-analysis by Kemp et al. (32) [for tricyclic antidepressant (TCA) only], treatment response is seldomly explored in association with HR and antidepressant medication. Few studies have specifically investigated the effect of HR/HRV with a link to treatment response: Hartmann et al. (11) and Park et al. (13) have found an increase of HF-HRV in patients responding to treatment, and Balogh et al. (10) have found higher HRV indexes (SDNN, RMSSD) particularly in responders to non-tricyclic antidepressant medication. However, others have found no effect or even a decrease of HRV parameters (RMSSD, HF-HRV, and LF-HRV) in response to pharmacological treatment (3, 15, 16) and electroconvulsive therapy (14). Yet, most studies have used relatively small sample sizes, and the change of covariates linked to antidepressant treatment (i.e., weight gain or enhanced activity) can play important moderating roles of this effect. Future research should be conducted as large-scale projects with emphasis on clinical characteristics and change of covariates.

## Limitations

This study has a number of limitations. First, the small number of participants does not allow for drawing robust conclusions based on the results or for extrapolating to the general population. However, the extremely high classification accuracy achieved in this small sample does support our model for classification approaches, although only with certainty for treatment-resistant depressed patients as assessed here. Furthermore, others have established chronotype as an important factor in circadian rhythm analyses. The assessment of this variable is missing here. Importantly, it was revealed that increased HR may be a consequence of antidepressant medication, in particular TCAs (47). Here, we assessed treatment-resistant depressed patients who have been treated with various antidepressant medications over several years, and all individuals tested were currently taking one or more antidepressants, including those known to have an impact on cardiovascular factors. The found alterations may therefore also be due to treatment effects rather than depression diagnosis *per se* or could be specific to treatment-resistant patients and thus cannot be generalized to all depressed patients. Furthermore, we also included three bipolar patients who, although they did not have a recent (hypo)manic episode, might affect the results. Since our data do not make it possible to control for antidepressant effects, larger studies in unmedicated patients are needed. In addition, patients in the ketamine group may have suspected to be in the verum group, since saline solution should not provoke any side effects. An active comparator like midazolam should be used in future studies. Lastly, since multiple doses of ketamine may show better response, future studies should use ketamine in a multiple-dose design.

## Conclusion

We confirm the value of HR/HRV as a trait marker for MDD: Increased HR and decreased HRV are striking hallmarks of depression, throughout both the day and night, with HR at 2–3 a.m. achieving excellent classification accuracy (90.6%). This might constitute a very reliable and easily accessible biomarker for depression. However, while these indexes may have some predictive potential for treatment response, we cannot confirm its relevance as a state marker for depression when using a single infusion of ketamine.

## Data Availability Statement

The data supporting this article is available under: https://data.mendeley.com/datasets/md4xp9xck6/1.

## Ethics Statement

The studies involving human participants were reviewed and approved by Ethics Committee Research UZ/KU Leuven. The patients/participants provided their written informed consent to participate in this study.

## Author Contributions

CS designed the study, wrote the protocol, recruited participants, performed analyses and literature search, and wrote the manuscript. EL designed and performed the analyses and helped with manuscript preparation. WD and CVH provided assistance with the wearables used during the trial and during data analysis. OC, VC, and MM helped with protocol design, participant recruitment, as well as manuscript correction. EV and SC helped with the conception and writing of the protocol, provided counsel on statistical analysis and manuscript preparation, and provided supervision. All authors have actively contributed to and have approved the final manuscript.

## Conflict of Interest

SC has a research collaboration with imec Belgium. WR and CVH are affiliated to imec Belgium. MM has received research funding from Janssen-Cilag Belgium, Takeda Pharmaceuticals Japan, and Lundbeck Belgium. The remaining authors declare that the research was conducted in the absence of any commercial or financial relationships that could be construed as a potential conflict of interest.

## Publisher's Note

All claims expressed in this article are solely those of the authors and do not necessarily represent those of their affiliated organizations, or those of the publisher, the editors and the reviewers. Any product that may be evaluated in this article, or claim that may be made by its manufacturer, is not guaranteed or endorsed by the publisher.

## References

[1] Alvares GA Quintana DS Hickie IB Guastella AJ. Autonomic nervous system dysfunction in psychiatric disorders and the impact of psychotropic medications: a systematic review and meta-analysis. J Psychiatry Neurosci. (2016) 41:89–104. 10.1503/jpn.140217 26447819 PMC4764485

[2] Byun S Kim AY Jang EH Kim S Choi KW Yu HY. Detection of major depressive disorder from linear and nonlinear heart rate variability features during mental task protocol. Comput Biol Med. (2019) 112:103381. 10.1016/j.compbiomed.2019.103381 31404718

[3] Glassman AH Bigger JT Gaffney M Van Zyl LT. Heart rate variability in acute coronary syndrome patients with major depression: influence of sertraline and mood improvement. Arch Gen Psychiatry. (2007) 64:1025–31. 10.1001/archpsyc.64.9.1025 17768267

[4] Iverson GL Gaetz MB Rzempoluck EJ McLean P Linden W Remick R. A new potential marker for abnormal cardiac physiology in depression. J Behav Med. (2005) 28:507–11. 10.1007/s10865-005-9022-7 16222413

[5] Stampfer HG. The relationship between psychiatric illness and the circadian pattern of heart rate. Aust N Z J Psychiatry. (1998) 32:187–98. 10.3109/00048679809062728 9588297

[6] Taillard J Lemoine P Boule P Drogue M Mouret J. Sleep and heart rate circadian rhythm in depression: the necessity to separate. Chronobiol Int. (1993) 10:63–72. 10.3109/07420529309064483 8443845

[7] Vaccarino V Badimon L Bremner JD Cenko E Cubedo J Dorobantu M. Depression and coronary heart disease: 2018 position paper of the ESC working group on coronary pathophysiology and microcirculation Eur Heart J. (2019) 41:1687–96. 10.1093/eurheartj/ehy913 30698764 PMC10941327

[8] Germain A Kupfer DJ. Circadian rhythm disturbances in depression. Hum Psychopharmacol. (2008) 23:571–85. 10.1002/hup.964 18680211 PMC2612129

[9] Bylsma LM Salomon K Taylor-Clift A Morris BH Rottenberg J. RSA reactivity in current and remitted major depressive disorder. Psychosom Med. (2014) 76:66–73. 10.1097/PSY.0000000000000019 24367127 PMC3906687

[10] Balogh S Fitzpatrick DF Hendricks SE Paige SR. Increases in heart rate variability with successful treatment in patients with major depressive disorder. Psychopharmacol Bull. (1993) 29:201–6. 8290666

[11] Hartmann R Schmidt FM Sander C Hegerl U. Heart rate variability as indicator of clinical state in depression. Front Psychiatry. (2018) 9:735. 10.3389/fpsyt.2018.00735 30705641 PMC6344433

[12] Karpyak VM Rasmussen KG Hammill SC Mrazek DA. Changes in heart rate variability in response to treatment with electroconvulsive therapy. J ECT. (2004) 20:81–8. 10.1097/00124509-200406000-00002 15167423

[13] Park SW Lee JH Kim J Suh S Lee MS. Changes in heart rate variability in first-episode drug-naïve adolescents with major depressive disorder: a 12-week prospective study. J Affect Disord. (2018) 238, 250–255. 10.1016/j.jad.2018.05.068 29890452

[14] Bozkurt A Barcin C Isintas M Ak M Erdem M Ozmenler KN. Changes in heart rate variability before and after ECT in the treatment of resistant major depressive disorder. Isr J Psychiatry Relat Sci. (2013) 50:40–6. 24029110

[15] Brunoni AR Kemp AH Dantas EM Goulart AC Nunes MA Boggio PS. Heart rate variability is a trait marker of major depressive disorder: evidence from the sertraline vs. electric current therapy to treat depression clinical study. Int J Neuropsychopharmacol. (2013) 16:1937–49. 10.1017/S1461145713000497 23759172

[16] Hage B Sinacore J Heilman K Porges SW Halaris A. Heart rate variability predicts treatment outcome in major depression. J Psychiatry Brain Sci. (2017) 2:1. 10.20900/jpbs.20170017

[17] Saad M Ray LB Bujaki B Parvaresh A Palamarchuk I De Koninck J. Using heart rate profiles during sleep as a biomarker of depression. BMC Psychiatry. (2019) 19:168. 10.1186/s12888-019-2152-1 31174510 PMC6554996

[18] Machado-Vieira R Baumann J Wheeler-Castillo C Latov D Henter ID Salvadore G. The timing of antidepressant effects: a comparison of diverse pharmacological and somatic treatments. Pharmaceuticals. (2010) 3:19–41. 10.3390/ph3010019 27713241 PMC3991019

[19] Serretti A Mandelli L. Antidepressants and body weight: a comprehensive review and meta-analysis. J Clin Psychiatry. (2010) 71:1259–72. 10.4088/JCP.09r05346blu 21062615

[20] Kishimoto T Chawla J Hagi K Zarate C Kane J Bauer M. Single-dose infusion ketamine and non-ketamine N-methyl-d-aspartate receptor antagonists for unipolar and bipolar depression: a meta-analysis of efficacy, safety and time trajectories. Psychol Med. (2016) 46:1459–72. 10.1017/S0033291716000064 26867988 PMC5116384

[21] Blier P Blier J. Ketamine: clinical studies in treatment-resistant depressive disorders. In: Mathew SJ Zarate JCA editors, Ketamine for Treatment-Resistant Depression: The First Decade of Progress. Cham: Springer International Publishing (2016). p. 31–42. 10.1007/978-3-319-42925-0_3

[22] Lecrubier Y Sheehan D Hergueta T Weiller E. The mini international neuropsychiatric interview. Eur Psychiatry. (1998) 13:198s. 10.1016/S0924-9338(97)86748-X 19698595

[23] Zimmerman M Martinez JH Young D Chelminski I Dalrymple K. Severity classification on the Hamilton depression rating scale. J Affect Disord. (2013) 150:384–8. 10.1016/j.jad.2013.04.028 23759278

[24] Irwin SA Iglewicz A Nelesen RA Lo JY Carr CH Romero SD. Daily oral ketamine for the treatment of depression and anxiety in patients receiving hospice care: a 28-day open-label proof-of-concept trial. J Palliat Med. (2013) 16:958–65. 10.1089/jpm.2012.0617 23805864 PMC3717203

[25] Berman RM Cappiello A Anand A Oren DA Heninger GR Charney DS. Antidepressant effects of ketamine in depressed patients. Biol Psychiatry. (2000) 47:351–4. 10.1016/S0006-3223(99)00230-9 10686270

[26] Heart rate variability: standards of measurement physiological interpretation and clinical use. Task Force of the European Society of Cardiology and the North American Society of Pacing and Electrophysiology. Circulation. (1996) 93:1043–65. 10.1111/j.1542-474X.1996.tb00275.x 8598068

[27] Orphanidou C Bonnici T Charlton P Clifton D Vallance D Tarassenko L. Signal-quality indices for the electrocardiogram and photoplethysmogram: derivation and applications to wireless monitoring. IEEE J Biomed Health Inform. (2014) 19:832–8. 10.1109/JBHI.2014.2338351 25069129

[28] Smets E Velazquez ER Schiavone G Chakroun I D'Hondt E De Raedt W. Large-scale wearable data reveal digital phenotypes for daily-life stress detection. npj Digit. Med. (2018) 1:67. 10.1038/s41746-018-0074-9 31304344 PMC6550211

[29] Cavallari JM Fang SC Mittleman MA Christiani DC. Circadian variation of heart rate variability among welders. Occup Environ Med. (2010) 67:717–9. 10.1136/oem.2010.055210 20798005 PMC3068835

[30] Max Kuhn (2021). caret: Classification and Regression Training. R package version 6.0-88. Available online at:https://CRAN.R-project.org/package=caret

[31] Green P MacLeod CJ. SIMR: an R package for power analysis of generalized linear mixed models by simulation. Methods Ecol Evol. (2016) 7:493–8. 10.1111/2041-210X.12504

[32] Kemp AH Quintana DS Gray MA Felmingham KL Brown K Gatt JM. Impact of depression and antidepressant treatment on heart rate variability: a review and meta-analysis. Biol Psychiatry. (2010) 67:1067–74. 10.1016/j.biopsych.2009.12.012 20138254

[33] Hamilton JL Alloy LB. Atypical reactivity of heart rate variability to stress and depression across development: systematic review of the literature and directions for future research. Clin Psychol Rev. (2016). 50:67–79. 10.1016/j.cpr.2016.09.003 27697746 PMC5233715

[34] Gaetz M Iverson GL Rzempoluck EJ Remick R McLean P Linden W. Self-organizing neural network analyses of cardiac data in depression. Neuropsychobiology. (2004) 49:30–7. 10.1159/000075336 14730198

[35] Lahmeyer HW Bellur SN. Cardiac regulation and depression. J Psychiatr Res. (1987) 21:1–6. 10.1016/0022-3956(87)90004-5 3560004

[36] Kuang D Yang R Chen X Lao G Wu F Huang X. Depression recognition according to heart rate variability using Bayesian Networks. J Psychiatr Res. (2017) 95:282–7. 10.1016/j.jpsychires.2017.09.012 28926794

[37] Guo Y-F Stein PK. Circadian rhythm in the cardiovascular system: chronocardiology. Am Heart J. (2003) 145:779–86. 10.1016/S0002-8703(02)94797-6 12766733

[38] Liebe T Li S Lord A Colic L Krause AL Batra A. Factors influencing the cardiovascular response to subanesthetic ketamine: a randomized, placebo-controlled trial. Int J Neuropsychopharmacol. (2017) 20:909–18. 10.1093/ijnp/pyx055 29099972 PMC5737852

[39] Hergovich N Singer E Agneter E Eichler HG Graselli U Simhandl C. Comparison of the effects of ketamine and memantine on prolactin and cortisol release in men: a randomized, double-blind, placebo-controlled trial. Neuropsychopharmacology. (2001) 24:590–3. 10.1016/S0893-133X(00)00194-9 11282259

[40] Penttilä J Mäenpää M Laitio T Långsjö J Hinkka S Scheinin H. Subanaesthetic doses of ketamine impair cardiac parasympathetic regulation. Eur J Anaesthesiol. (2005) 22:808–10. 10.1017/S0265021505271326 16211790

[41] Durieux ME. Inhibition by ketamine of muscarinic acetylcholine receptor function. Anesth Analg. (1995) 81:57–62. 10.1097/00000539-199507000-00012 7598283

[42] Komatsu T Singh PK Kimura T Nishiwaki K Bando K Shimada Y. Differential effects of ketamine and midazolam on heart rate variability. Canad J Anaesth. (1995) 42:1003–9. 10.1007/BF03011073 8590488

[43] Meyer T Brunovsky M Horacek J Novak T Andrashko V Seifritz E. Predictive value of heart rate in treatment of major depression with ketamine in two controlled trials. Clin Neurophysiol. (2021) 132:1339–46. 10.1016/j.clinph.2021.01.030 33888426

[44] Choi KW Jang EH Kim AY Fava M Mischoulon D Papakostas GI. Heart rate variability for treatment response between patients with major depressive disorder versus panic disorder: a 12-week follow-up study. J Affect Disord. (2019) 246:157–65. 10.1016/j.jad.2018.12.048 30583140

[45] Kircanski K Williams LM Gotlib IH. Heart rate variability as a biomarker of anxious depression response to antidepressant medication. Depress Anxiety. (2019) 36:63–71. 10.1002/da.22843 30311742 PMC6318007

[46] Licht CM de Geus EJ van Dyck R Penninx BW. Longitudinal evidence for unfavorable effects of antidepressants on heart rate variability. Biol Psychiatry. (2010) 68:861–8. 10.1016/j.biopsych.2010.06.032 20843507

[47] Burckhardt D Raeder E Müller V Imhof P Neubauer H. Cardiovascular effects of tricyclic and tetracyclic antidepressants. JAMA J Am Med Assoc. (1978) 239:213–6. 10.1001/jama.1978.03280300045019 579392

