# Supplementary material for: Twenty-Four-Hour Heart Rate Is a Trait but Not State Marker for Depression in a Pilot Randomized Controlled Trial With a Single Infusion of Ketamine
Source: Front Psychiatry. 2021 Jul 29;12:696170. doi: 10.3389/fpsyt.2021.696170 (PMC8358607; doi:10.3389/fpsyt.2021.696170)
Supplement: Supplementary file 1 [file Data_Sheet_1.docx]

***Supplementary Material***

**Supplementary Figure 1. CONSORT 2010 Flow Diagram**

Lost to follow-up (give reasons) (n=0)
Discontinued intervention (give reasons) (n=0)

## Follow-Up

Allocated to intervention Ketamine (n=21)

 Received allocated intervention (n= 21)

 Did not receive allocated intervention (give reasons) (n= 0)

## Allocation

Randomized (n= 28)

Assessed for eligibility (n=29)


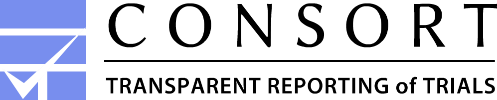


## Enrolment

Excluded (n=1)

  Not meeting inclusion criteria (n= 1)

  Declined to participate (n= 0)

  Other reasons (n= 0)

Allocated to intervention Placebo (n=7)

 Received allocated intervention (n=7)

 Did not receive allocated intervention (give reasons) (n=0)

Lost to follow-up (give reasons) (n=0 )
Discontinued intervention (give reasons) (n=0)

Analysed (total n=12; baseline:n=12, postphase:n=11,no data:n=9)

Reasons for exclusion: ECG data missing

Baseline analysis: n=9 missing at pre and postphase

Post treatment: n=1 excluded because data was missing from post phase

## Analysis

Analysed (total n= 4, baseline: n=4, postphase n=2, no data n=3)

Reasons for exclusion: ECG data missing

Baseline analysis: n=3 missing data at pre and postphase

Post-treatment: n=2 excluded due to no data in the postphase

**B**

**A**


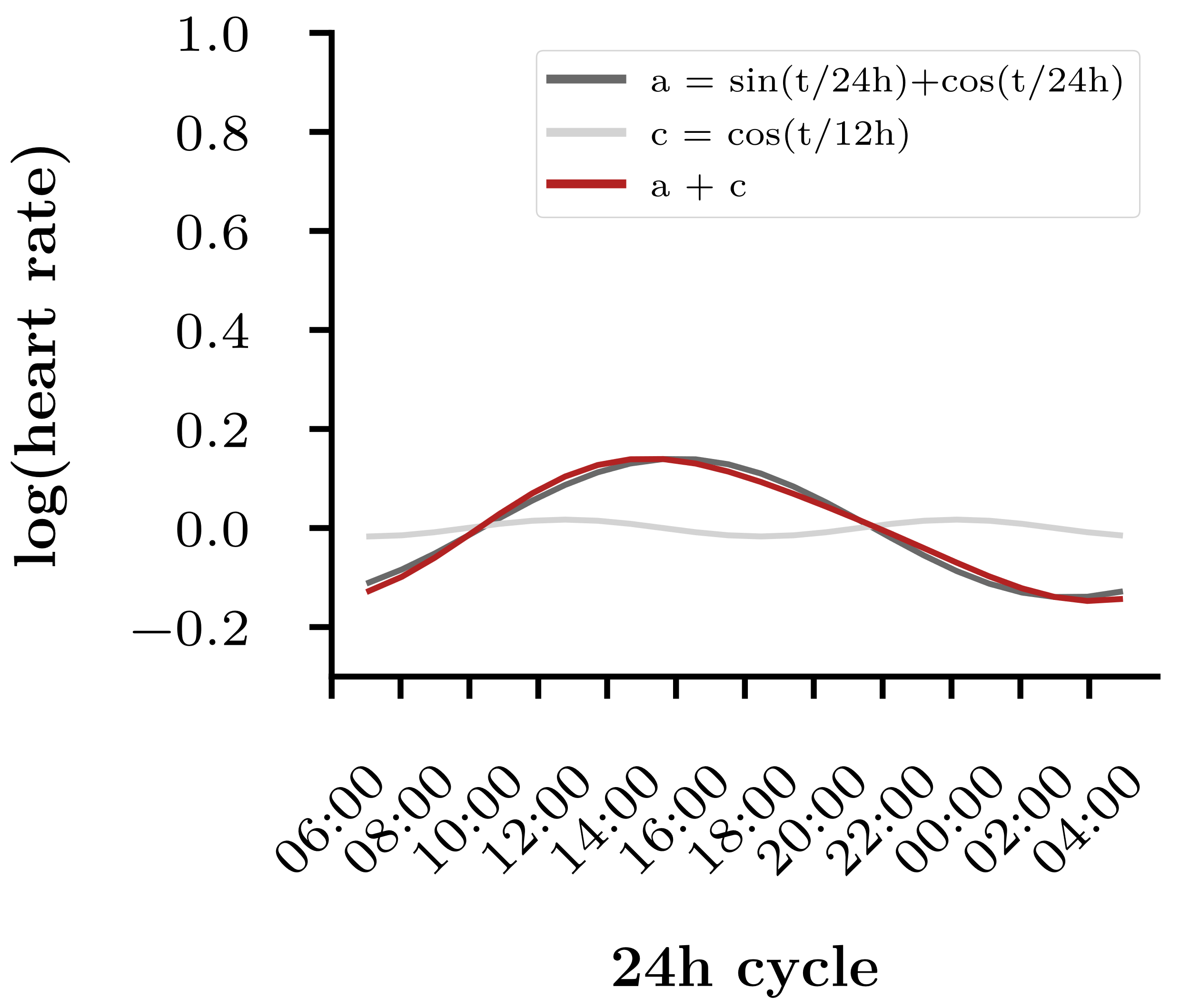

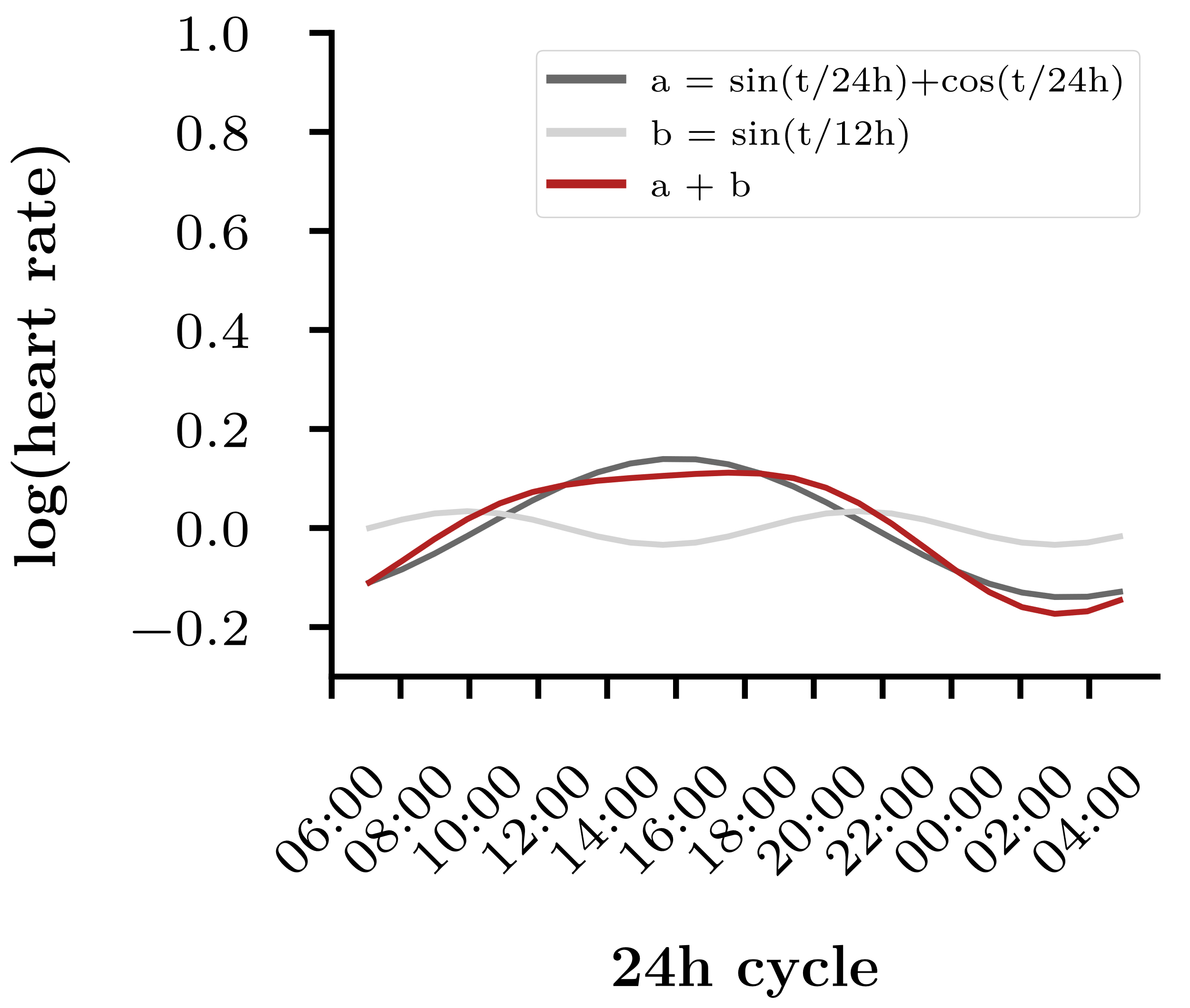


**Supplementary Figure 2.** *Demonstration of the separate harmonic regression terms introduced to fit the regression line for circadian variations at the example of HR.* **A**. Simulation of heart rate. The dark grey line shows the traditional *cosinor* variation introduced by 24hour cosines and sinus terms. The light grey line shows the effect of a 12hour sinus term added to the model. The red line demonstrates the combination of 12hour sinus term together with the traditional 24h cosines and sinus terms. **B.** Analog to A, this graph shows the modulation by adding a cosines 12-hour term to the 24 hour cosines and sinus terms, again showing a shift of the additive curve (cosines 12h with cosines and sinus for 24hours). **Supplementary** **Table 2** summarizes the specific effect for the curves.


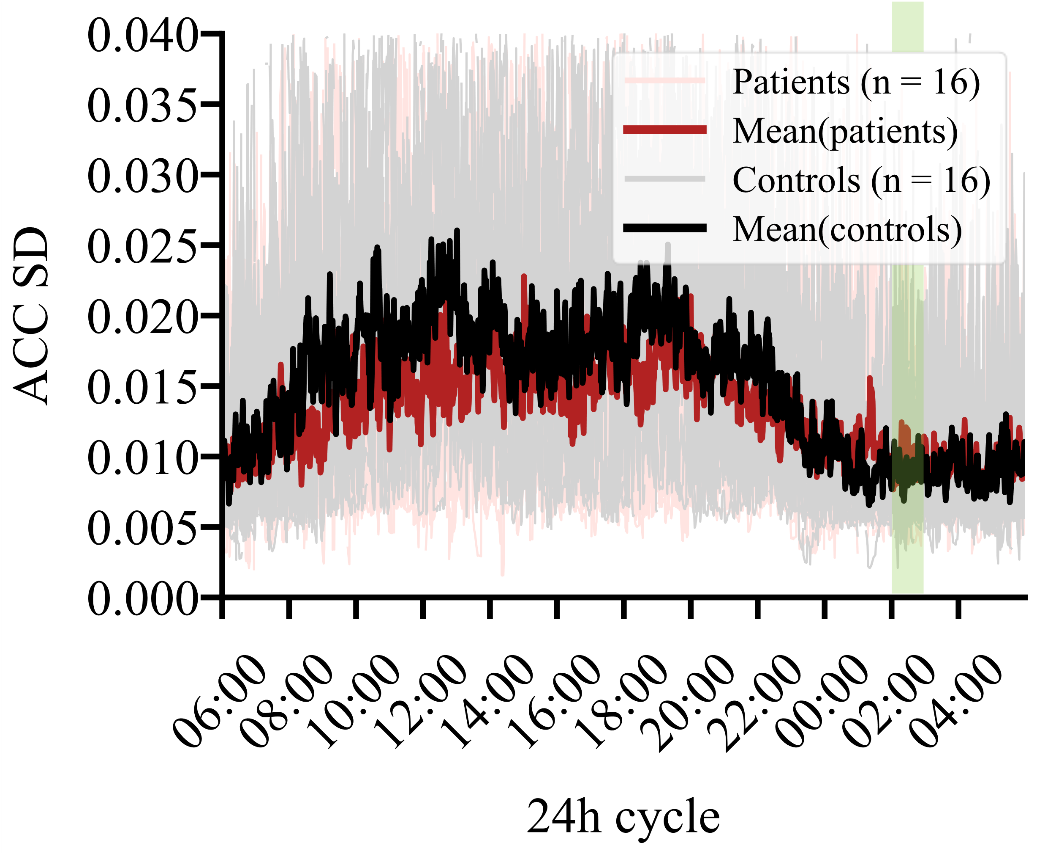


**Supplementary Figure 3.**  *Activity levels of patients with MDD and healthy controls.* The green surface marks virtually overlapping levels of lowest activity during the night (2-3 AM) used for classification of patients.

**Supplementary Table 1A.** *Concomitant medication for the responders and non-responders.* This table summarizes data for the 16 patients regardless of whether they received placebo/ketamine or whether they had post-hoc data available.

| **AD medication** | **Responders** | **Non-responders** |
| --- | --- | --- |
| SSRI/SNRI/SARI | 6/8 (75%) | 5/8 (62.5%) |
| TCA/TeCA | 3/8 (37.5%) | 2/8 (25%) |
| Benzodiazepines | 2/8 (25%) | 4/8 (50%) |
| Other | 1/8 (12.5%) | 3/8 (37.5%) |

**Supplementary Table 1B.** Concomitant medication per participant. A "1" indicates that the person is taking a certain class of medication. The tab "other" medication contains various medications including vitamin supplements.

| **Subject** | **Group** | **SSRI** | **SNRI** | **SARI** | **NDRI/NRI** | **Monoaminergic** | **TCA/Teca** | **Atypical** | **Benzo** | **Antipsychotic** | **Anticonvulsive** | **Contraceptive** | **Anti-inflammatory** | **Thyroid** | **Opiate** | **Other** |
| --- | --- | --- | --- | --- | --- | --- | --- | --- | --- | --- | --- | --- | --- | --- | --- | --- |
| 1 | Control | 0 | 0 | 0 | 0 | 0 | 0 | 0 | 0 | 0 | 0 | 0 | 0 | 0 | 0 | 0 |
| 2 | Control | 0 | 0 | 0 | 0 | 0 | 0 | 0 | 0 | 0 | 0 | 0 | 0 | 0 | 0 | 0 |
| 3 | Control | 0 | 0 | 0 | 0 | 0 | 0 | 0 | 0 | 0 | 0 | 0 | 0 | 0 | 0 | 0 |
| 4 | Control | 0 | 0 | 0 | 0 | 0 | 0 | 0 | 0 | 0 | 0 | 1 | 0 | 0 | 0 | 0 |
| 5 | Control | 0 | 0 | 0 | 0 | 0 | 0 | 0 | 0 | 0 | 0 | 1 | 0 | 0 | 0 | 0 |
| 6 | Control | 0 | 0 | 0 | 0 | 0 | 0 | 0 | 0 | 0 | 0 | 0 | 0 | 0 | 0 | 0 |
| 7 | Control | 0 | 0 | 0 | 0 | 0 | 0 | 0 | 0 | 0 | 0 | 1 | 0 | 0 | 0 | 0 |
| 8 | Control | 0 | 0 | 0 | 0 | 0 | 0 | 0 | 0 | 0 | 0 | 1 | 0 | 0 | 0 | 0 |
| 9 | Control | 0 | 0 | 0 | 0 | 0 | 0 | 0 | 0 | 0 | 0 | 0 | 0 | 0 | 0 | 0 |
| 10 | Control | 0 | 0 | 0 | 0 | 0 | 0 | 0 | 0 | 0 | 0 | 0 | 0 | 0 | 0 | 0 |
| 11 | Control | 0 | 0 | 0 | 0 | 0 | 0 | 0 | 0 | 0 | 0 | 0 | 0 | 0 | 0 | 0 |
| 12 | Control | 0 | 0 | 0 | 0 | 0 | 0 | 0 | 0 | 0 | 0 | 1 | 0 | 0 | 0 | 0 |
| 13 | Control | 0 | 0 | 0 | 0 | 0 | 0 | 0 | 0 | 0 | 0 | 0 | 0 | 0 | 0 | 0 |
| 14 | Control | 0 | 0 | 0 | 0 | 0 | 0 | 0 | 1 | 0 | 0 | 0 | 0 | 0 | 0 | 0 |
| 15 | Control | 0 | 0 | 0 | 0 | 0 | 0 | 0 | 0 | 0 | 0 | 1 | 0 | 0 | 0 | 0 |
| 16 | Control | 0 | 0 | 0 | 0 | 0 | 0 | 0 | 0 | 0 | 0 | 0 | 0 | 0 | 0 | 1 |
| 17 | Patient | 0 | 1 | 0 | 0 | 0 | 0 | 0 | 0 | 0 | 1 | 1 | 0 | 0 | 0 | 0 |
| 18 | Patient | 0 | 1 | 0 | 0 | 0 | 0 | 0 | 1 | 0 | 1 | 1 | 0 | 0 | 0 | 1 |
| 19 | Patient | 0 | 1 | 0 | 0 | 0 | 0 | 1 | 0 | 0 | 0 | 0 | 0 | 1 | 0 | 1 |
| 20 | Patient | 0 | 1 | 0 | 0 | 0 | 0 | 1 | 0 | 0 | 1 | 0 | 0 | 1 | 0 | 0 |
| 21 | Patient | 0 | 1 | 0 | 0 | 0 | 0 | 0 | 0 | 0 | 0 | 1 | 0 | 0 | 0 | 0 |
| 22 | Patient | 1 | 0 | 0 | 1 | 0 | 0 | 0 | 0 | 0 | 0 | 0 | 0 | 0 | 0 | 0 |
| 23 | Patient | 1 | 1 | 1 | 0 | 0 | 0 | 1 | 0 | 0 | 0 | 1 | 0 | 0 | 0 | 0 |
| 24 | Patient | 0 | 1 | 0 | 0 | 0 | 1 | 1 | 0 | 0 | 0 | 0 | 0 | 0 | 0 | 0 |
| 25 | Patient | 0 | 0 | 0 | 0 | 0 | 1 | 1 | 0 | 0 | 0 | 0 | 0 | 0 | 0 | 1 |
| 26 | Patient | 0 | 0 | 0 | 0 | 0 | 1 | 0 | 1 | 0 | 0 | 0 | 0 | 0 | 1 | 1 |
| 27 | Patient | 1 | 0 | 0 | 1 | 0 | 0 | 0 | 1 | 0 | 0 | 0 | 0 | 0 | 0 | 1 |
| 28 | Patient | 1 | 0 | 1 | 1 | 0 | 0 | 1 | 0 | 0 | 0 | 0 | 0 | 0 | 0 | 1 |
| 29 | Patient | 0 | 0 | 0 | 0 | 0 | 0 | 0 | 0 | 1 | 0 | 0 | 0 | 0 | 0 | 0 |
| 30 | Patient | 0 | 0 | 0 | 0 | 0 | 1 | 0 | 1 | 1 | 0 | 0 | 0 | 0 | 0 | 1 |
| 31 | Patient | 0 | 0 | 0 | 0 | 0 | 0 | 0 | 1 | 1 | 0 | 0 | 0 | 0 | 0 | 1 |
| 32 | Patient | 1 | 0 | 0 | 0 | 0 | 1 | 0 | 1 | 1 | 1 | 0 | 0 | 0 | 0 | 1 |

**Supplementary Table 2.** *Description of the harmonic regression term modulation on physiological signals.* The estimates for sinus and cosines harmonic regression terms are derived from the regression model of the overall group (patients and controls) for illustration purposes.

| *Mathematical expression* | *Wave form and alterations* | *Result* |
| --- | --- | --- |
| *[-0,108*sin(*${2\pi}/{24h}$*)]*  *+ [-0.087*cos(*${2\pi}/{24h}$*)]* | - presents a maximum HR around 4PM and minimum HR around 3AM | Rise in heart rate until noon, followed by an intermediate decrease in the afternoon and a second elevation in the evening, with steep descend until 3AM |
| *+ [-0,033*sin (*${2\pi}/{12h}$*)]*  (Supplementary Figure 1A) | - flattens the maximum HR, resulting in a plateau from 10AM to 6PM. - shifts maximum HR towards evening - lowers the minimum HR |  |
| + [0.017*cos(${2\pi}/{12h}$)]]  (Supplementary Figure 1B) | - shifts the maximum HR towards the noon |  |

**Supplementary Information 1. Power calculations**

###############
# Power calculation for 60 participants
###############

library(lme4)
library(simr)

*## 1. Setting up a simulated dataframe for 60 participants (30 HC and 30 patients) with each three day of 24h data*

*# 60 subjects*
subnr = 60

*# three days*
l = 3*24
subj <- factor(1:subnr) # 60 subjects
subj_full <- rep(subj, each=l)

*# 30 HC and 30 patients*
group <- c("control", "depression")
group_full <- rep(rep(group, each=l),subnr/2)

*# Random sample with ages between 20 and 50 years old*
age <- sample(20:50,subnr, replace = T)
age_full <- rep(age, each=l)

*# Equal number of males and females in both groups*
sex <- c("M", "F", "F","M")
sex_full <- rep(rep(sex, each=l),subnr/4)

*# Random samples with BMI values between 19 and 30*
bmi <- sample(19:30,subnr, replace = T)
bmi_full <- rep(bmi, each=l)

*# Activity level between 0.01 and 0.06*
std_ACC <- sample(1:6,subnr, replace = T)/100
STD_ACC <- rep(std_ACC, l)

*# Circadian rhythm*
time = 1:24
time <- rep(time,3)
Hourblock <- rep(time, subnr)
wf1 = (sin(2*pi/24*(Hourblock)))
wf2 = (cos(2*pi/24*(Hourblock)))
wf3 = (sin(2*pi/12*(Hourblock)))
wf4 = (cos(2*pi/12*(Hourblock)))

*# Resulting dataframe*
covars <- data.frame(Subject=subj_full, Group=factor(group_full), Age=age_full, Sex = factor(sex_full), BMI = bmi_full, Activity = STD_ACC, wavef1 = wf1, wavef2 = wf2, wavef3 = wf3, wavef4 = wf4)

*### 2. Defining fixed effecs for Intercept, Group, Age, Sex, BMI, Activity, wavef1, wavef2, wavef3, wavef4, Group*wavef1, Group*wavef2, Group*wavef3, Group*wavef4*
fixed <- c(4,0.1, 0.1, 0.1, 0.1, 0.1,0.05, 0.05, 0.02, 0.02, 0.02, 0.02, 0.01, 0.01)

*## 3. Defining random intercepts for participants*rand <- list(0.01)

*## 4. Defining residual variance*
res <- 0.1

*## 5. Creating the model*model <- makeLmer(y~ Group +Age +Sex + BMI + Activity + wavef1 + wavef2 + wavef3 + wavef4 +Group:wavef1+Group:wavef2+Group:wavef3+Group:wavef4 +
 (1|Subject), fixef = fixed, VarCorr = rand, sigma = res, data = covars)

## 6. Power analysis
powerSim(model, test=fixed("Group"), nsim =200)

PowerSim(model, test=fixed("Group"), nsim =100)

Power for predictor 'Group', (95% confidence interval):

98.00% (92.96, 99.76)

Test: Likelihood ratio

Based on 100 simulations, (100 warnings, 0 errors)

alpha = 0.05, nrow = 4320

Time elapsed: 0 h 0 m 45 s

> powerSim(model, test=fixed("Group:wavef1"), nsim =100)

Power for predictor 'Group:wavef1', (95% confidence interval):

99.00% (94.55, 99.97)

Test: Kenward Roger (package pbkrtest)

Based on 100 simulations, (0 warnings, 0 errors)

alpha = 0.05, nrow = 4320

Time elapsed: 1 h 0 m 41 s

> powerSim(model, test=fixed("Group:wavef3"), nsim =100)

Power for predictor 'Group:wavef3', (95% confidence interval):

58.00% (47.71, 67.80)

Test: Kenward Roger (package pbkrtest)

Based on 100 simulations, (0 warnings, 0 errors)

alpha = 0.05, nrow = 4320

Time elapsed: 1 h 4 m 42 s

###############
# Power calculation for 32 participants
###############

library(lme4)
library(simr)

*## 1. Setting up a simulated dataframe for 32 participants (16 HC and 16 patients) with each three day of 24h data*

*# 32 subjects*
subnr = 32

*# three days*
l = 3*24
subj <- factor(1:subnr) # 32 subjects
subj_full <- rep(subj, each=l)

*# 16 HC and 16 patients*
group <- c("control", "depression")
group_full <- rep(rep(group, each=l),subnr/2)

*# Random sample with ages between 20 and 50 years old*
age <- sample(20:50,subnr, replace = T)
age_full <- rep(age, each=l)

*# Equal number of males and females in both groups*
sex <- c("M", "F", "F","M")
sex_full <- rep(rep(sex, each=l),subnr/4)

*# Random samples with BMI values between 19 and 30*
bmi <- sample(19:30,subnr, replace = T)
bmi_full <- rep(bmi, each=l)

*# Activity level between 0.01 and 0.06*
std_ACC <- sample(1:6,subnr, replace = T)/100
STD_ACC <- rep(std_ACC, l)

*# Circadian rhythm*
time = 1:24
time <- rep(time,3)
Hourblock <- rep(time, subnr)
wf1 = (sin(2*pi/24*(Hourblock)))
wf2 = (cos(2*pi/24*(Hourblock)))
wf3 = (sin(2*pi/12*(Hourblock)))
wf4 = (cos(2*pi/12*(Hourblock)))

*# Resulting dataframe*
covars <- data.frame(Subject=subj_full, Group=factor(group_full), Age=age_full, Sex = factor(sex_full), BMI = bmi_full, Activity = STD_ACC, wavef1 = wf1, wavef2 = wf2, wavef3 = wf3, wavef4 = wf4)

*### 2. Defining fixed effecs for Intercept, Group, Age, Sex, BMI, Activity, wavef1, wavef2, wavef3, wavef4, Group*wavef1, Group*wavef2, Group*wavef3, Group*wavef4*
fixed <- c(4,0.1, 0.1, 0.1, 0.1, 0.1,0.05, 0.05, 0.02, 0.02, 0.02, 0.02, 0.01, 0.01)

*## 3. Defining random intercepts for participants*rand <- list(0.01)

*## 4. Defining residual variance*
res <- 0.1

*## 5. Creating the model*model <- makeLmer(y~ Group +Age +Sex + BMI + Activity + wavef1 + wavef2 + wavef3 + wavef4 +Group:wavef1+Group:wavef2+Group:wavef3+Group:wavef4 +
 (1|Subject), fixef = fixed, VarCorr = rand, sigma = res, data = covars)

## 6. Power analysis
>powerSim(model, test=fixed("Group"), nsim =100)

Power for predictor 'Group', (95% confidence interval):

80.00% (70.82, 87.33)

Test: Likelihood ratio

Based on 100 simulations, (100 warnings, 0 errors)

alpha = 0.05, nrow = 2304

Time elapsed: 0 h 0 m 28 s

> powerSim(model, test=fixed("Group:wavef1"), nsim =100)

Power for predictor 'Group:wavef1', (95% confidence interval):

95.00% (88.72, 98.36)

Test: Kenward Roger (package pbkrtest)

Based on 100 simulations, (0 warnings, 0 errors)

alpha = 0.05, nrow = 2304

Time elapsed: 0 h 10 m 54 s

> powerSim(model, test=fixed("Group:wavef3"), nsim =100)

Power for predictor 'Group:wavef3', (95% confidence interval):

45.00% (35.03, 55.27)

Test: Kenward Roger (package pbkrtest)

Based on 100 simulations, (0 warnings, 0 errors)

alpha = 0.05, nrow = 2304

Time elapsed: 0 h 11 m 39 s
